# Total knee arthroplasty survivorship and outcomes in young patients: a review of the literature and 40-year update to a longitudinal study

**DOI:** 10.1007/s00402-024-05198-5

**Published:** 2024-03-04

**Authors:** Victoria E. Bergstein, Aaron I. Weinblatt, Walter L. Taylor, William J. Long

**Affiliations:** 1 Department of Adult Reconstruction and Joint Replacement, Hospital for Special Surgery, New York, NY USA; 2 Johns Hopkins University School of Medicine, Baltimore, MD USA

**Keywords:** Total knee arthroplasty, Young patients, Osteoarthritis, Functional outcomes, Implant survivorship

## Abstract

**Introduction:**

Growing numbers of younger patients are electing to undergo total knee arthroplasty (TKA) for end-stage osteoarthritis. The purpose of this study was to compare established literature regarding TKA outcomes in patients under age 55, to data from an ongoing longitudinal young patient cohort curated by our study group. Further, we aimed to provide a novel update on survivorship at 40 years post-TKA from our longitudinal cohort.

**Methods:**

A literature search was conducted using the electronic databases PubMed, Embase, and Cochrane Library, using terms related to TKA, patients under age 55, and osteoarthritis. Demographic and outcome data were extracted from all studies that met the inclusion criteria. Data were divided into the “longitudinal study (LS) group,” and the “literature review (LR) group” based on the patient population of the study from which it came.

**Results:**

After screening, 10 studies met the inclusion criteria; 6 studies comprised the LR group, and 4 studies comprised the LS group. 2613 TKAs were performed among the LR group, and 114 TKAs were longitudinally followed in the LS group. The mean patient ages of the LR and LS groups were 46.1 and 51, respectively. Mean follow-up was 10.1 years for the LR group. Mean postoperative range of motion was 113.6° and 114.5° for the LR and LS groups, respectively. All-cause survivorship reported at 10 years or less ranged from 90.6% to 99.0%. The LS cohort studies reported survivorship ranges of 70.1–70.6% and 52.1–65.3% at 30 and 40 years, respectively.

**Conclusions:**

Young TKA patients demonstrated improved functionality at each follow-up time point assessed. Survivorship decreased with increasing lengths of follow-up, ultimately ranging from 52.1–65.3% at 40 years post-TKA. The paucity of literature on long-term TKA outcomes in this patient population reinforces the necessity of further research on this topic.

## Introduction

Total knee arthroplasty (TKA) is a consistently successful and in-demand procedure in the United States, with an estimated 800,000 procedures performed annually [1]. The combination of the procedure’s positive outcomes and the United States’ aging population has led physicians to forecast an exponential increase in TKAs over the coming decades, with projections of over 3.4 million annual TKAs being performed by 2040 [2]. The most common indication for TKA is osteoarthritis, a condition that can cause marked disability [3]. Studies have shown that the incidence of osteoarthritis increases with age, and then plateaus around age 70 [4]. This incidence is steadily increasing as the population ages, and as obesity becomes more prevalent, creating a disease burden for which the incidence of TKA is expected to increase in response [3].

Though the average age of a TKA patient has been reported to fall around 67 years, the incidence of TKA in younger patients is also quickly rising [4]. There are varieties of reasons for which TKA in younger patients is both encouraged and discouraged. The efficacious outcomes of TKA are appealing when compared to long-term non-operative treatment like physical therapy and corticosteroid injections which usually do not offer sustained relief [5]. Conversely, while TKA has been shown to have long-lasting survivorship, young, more active patients are more likely to wear out the implant during their lifetime, increasing their risk for revision [6–10]. For example, in a review of national-level data, the Australian Joint Registry reported a survivorship rate of 91.2% at 10 years among primary TKA patients under 55, compared to a range of 94.1–97.1% at 10 years for patients older than 55 [11].

Our research group has conducted a longitudinal study over the past 40 years, investigating TKA survivorship in patients under the age of 55. The purpose of this study is to compare established literature regarding TKA outcomes in young patients to those reporting on our longitudinal patient cohort, reported at 15 and 30 years. Notably, we will also report ongoing research on this patient cohort that includes novel, unpublished data being reported 40 years post-TKA. Our primary outcome measures are TKA functional outcomes, survivorship, risk factors for revision, and revision rates among patients 55 years of age and younger, for the primary indication of osteoarthritis.

## Methods

### Literature search

A literature search was conducted using the electronic databases PubMed, Embase, and Cochrane Library. Search terms included “total knee arthroplast*” or “total knee replacement,” and “young patient” or “young” or “under 55” or “55 years,” and “osteoarthritis.” A general keyword search using these terms was performed first, and then the reviewing authors manually looked through the search results and selected articles that actually contained the search terms. Reference lists from each article were screened, and a manual search through PubMed and Google Scholar was also performed to identify any missing publications. References were managed and subsequently screened with the Covidence systematic review software (Veritas Health Innovation, Melbourne, Australia. Available at covidence.org).

### Inclusion and exclusion criteria

The goal of the literature search was to find studies with study populations similar to the cohort initially described in the 1997 paper by Diduch et al. [12]. As such, studies met the inclusion criteria if they reported on patient outcomes of primary TKA in patients 55 years or younger, and if at least 80% of the patient cohort had a preoperative diagnosis of osteoarthritis. Articles were excluded if patients underwent revision TKA or unicompartmental knee replacements, if over 20% of the patient cohort had preoperative diagnoses of rheumatoid arthritis or inflammatory arthropathy, if the patient cohort included patients older than age 55, or if the mean age of the patient cohort was not between 40 and 55. Studies were excluded if the primary outcome measure was a comparison between novel and standard implants.

### Data extraction

Studies were identified and screened by two authors (V.B. and A.W.). Studies determined to meet all inclusion criteria were listed in an Excel spreadsheet and data was extracted. Extracted demographic data included average age of the patient cohort, study period time range, mean follow-up, preoperative diagnosis, number of TKAs, implant type, fixation type (cemented versus uncemented), and cruciate-retaining or posterior-stabilized design. Extracted outcome data included preoperative and postoperative Knee Society (KS) clinical and functional scores, preoperative and postoperative range of motion, survivorship, revision rate, risk factors for revision, revision etiologies, and radiolucency. Data were divided into groups based on the origin of the study from which it came; the four studies reporting on the same patient cohort are henceforth referred to as the “longitudinal study (LS) group,” and the six studies with differing cohorts are referred to as the “literature review (LR) group.” Data was collected from each group to determine mean values and improvements, and subsequently, the data was combined across all studies to determine average outcome scores.

### 40-Year follow-up

The 40-year follow-up was conducted by contacting all patients included in the initial cohort described by Diduch et al. via the phone number they had on file [12]. This element of the study was approved by our institution’s Institutional Review Board (IRB #2021-0990). If this contact information was unavailable to us in our prior study documents, we attempted to gather this information through our institution’s electronic medical record. If the patient’s contact information was not found through either of these methods, we considered the patient’s knee status to be unknown.

If the patient was listed as revised in the data from our previous follow-up report performed at 30 years, this information was carried over into our most recent 40-year follow-up to calculate cumulative survivorship [13]. Similarly, if the patient was listed as unrevised in the 30-year follow-up, but our team was unable to retrieve an update on them, their status was considered unknown but accounted for in the calculation of the upper limit of survivorship at 40 years. The lower limit for survivorship at 40 years was calculated as the percentage of patients revised out of the total cohort with known revised or unrevised status.

## Results

### Search results

The initial literature search, which was a combination of electronic and manual identification of articles, yielded 103 studies, from which 11 duplicates were removed. The remaining 92 studies were evaluated, of which 61 were determined irrelevant and removed. 27 full-text articles were assessed using the determined eligibility criteria, and 10 papers were ultimately included in the present study, 4 of which were part of the LS group curated by Diduch et al. most recently reviewed by Long et al. [12, 13].

### Patient demographics

Between the 6 included studies in the LR group, 2613 TKAs were performed. The mean patient age across these studies was 46.1, and mean follow-up was 10.1 years. 100% of patients had preoperative diagnoses of osteoarthritis or posttraumatic osteoarthritis in 2 of the 6 studies, and the proportion of patients with osteoarthritis was 80% or greater in the remaining studies. Two studies used only cemented fixation, 2 studies used both cemented and uncemented fixation, and 2 studies used cemented, uncemented, and hybrid fixation. While none of the studies exclusively used posterior-stabilized (PS) designs, 2 studies exclusively used cruciate-retaining (CR) designs, and 4 studies used a combination of both.

There were four studies in the LS group, all of which reported upon the same patient population. The mean patient age was 51, with a range of 22 to 55, and all patients had preoperative diagnoses of osteoarthritis or post-traumatic osteoarthritis. Follow-ups in the four studies were performed at averages of 8 years, 10.5 years, and two studies were performed at an average of 25.1 years, respectively. All of the TKAs in this group were posterior-stabilized and cemented. Patient demographics for both the literature review group and the longitudinal study group are summarized in Table 1.

**Table 1:** Patient cohort demographics

Study	Number of TKAs	Average age (Range)	Mean follow-up (Years)	Fixation type	Cruciate-retaining / posterior-stabilized
Literature review cohort					
Aujla et al. [13]	1224	< 55	10.8	Cemented, hybrid, uncemented	CR and PS
Odland et al.[6]	67	49 (28–55)	11.2	Cemented	CR and PS
Keeney et al. [14]	908	< 55	5 to 18	Cemented, uncemented	CR and PS
Goh et al. [15]	136	47 (30–50)	6.7	Cemented	CR and PS
Karas et al. [10]	248	46 (26–49)	13	Cemented, uncemented	CR
Mont et al. [16]	30	43 (31–50)	7.2	Cemented, hybrid, uncemented	CR
Longitudinal study cohort					
Diduch et al. [11]	114	51 (22–25)	8	Cemented	PS
Old et al. [17]	114	50	10.5	Cemented	PS
Long et al. [12]	107	51 (22–55)	25.1	Cemented	PS
Camus et al. [7]	114	51 (22–55)	25.1	Cemented	PS

Of the 84 patients (108 knees) included in the LS cohort’s 30-year follow-up in 2012, we were able to obtain data from 57 patients (71 knees) at the current stage of our 2022 40-year follow-up. 29 patients (42 knees) were confirmed to have died at this stage of the follow-up. 31 patients (43 knees) were lost to follow-up over the course of the 40-year study.

### Functional outcomes

All outcomes are detailed in Table 2. Of the 6 studies in the LR cohort that reported postoperative range of motion (ROM), the mean ROM was 113.6°. 5 of the LR studies provided preoperative comparative ROM values, in which the average ROM improvement in those studies was 7.9° using the latest ROM measurement available. 2 of the LS cohort studies reported postoperative ROM, with a mean of 114.5°. Preoperative ROM was not reported in the LS cohort, so improvement of ROM could not be obtained.

**Table 2:** Young patient outcomes following TKA

Study	Preoperative to postoperative range of motion	Postoperative knee society clinical score (Improvement)	Postoperative knee society functional score (Improvement)	Survivorship	Revision rate
Literature review cohort					
Aujla et al.	108.8° → 111.7°	89.7 (54.4)	81.1 (45.5)	98.2% at 10 years	5.40%
Odland et al.	106° → 114°	91.2 (39.0)	79.5 (26.4)	–	16.40%
Keeney et al.	102° → 109°	90.9 (47)	81.6 (37)	90.6–99% at 10 years 85–96.5% at 20 years	5.50%
Goh et al.	106° → 111.2°	83.1 (47.9)	78.8 (26)	97.8% at 6.7 years	2.20%
Karas et al.	100.2° → 116.8°	85.7 (41.3)	79.0 (29.3)	83.9% at 13 years	10.90%
Mont et al.	118°^a^	89 (40)	70^a^	–	3.30%
Longitudinal study cohort					
Diduch et al.	–	84^a^	89^a^	94% at 8 years	6.10%
Old et al.	119°^a^	89.0^a^	75.6^a^	–	21.90%
Long et al.	110°^a^	87.4^a^	62.1^a^	70.1% at 25.1 years	23.40%
Camus et al.	–	–	–	70.6% at 25.1 years	–
Unpublished 40-Year data	–	–	–	52.1–65.3% at 40 years	–

^a^Reported scores are postoperative only

The average postoperative KS clinical and functional scores between the 9 studies that reported them were 87.8 and 77.4, respectively. Both scoring systems have a maximum of 100 points—this upper bound describing greater than 125 degrees of flexion and a stable, painless knee [14]. Of the 6 studies that also reported preoperative clinical KS scores and 5 studies that reported preoperative functional KS scores, the mean improvements in scores were 44.9 and 33.2, respectively.

In the LS cohort, Long et al. stratified patient outcomes on the basis of a modified Charnley classification, where category A consisted of a unilateral or successful bilateral total knee arthroplasty without symptoms in the contralateral knee, category B experienced symptoms in the contralateral knee, and category C had associated medical conditions that limited function [13, 15]. Category A patients were found to have no differences in function from 15 to 30 years post-TKA [13]. 72.2% of the total cohort had improved activity scores at their 25-year follow-up, and 25% of the cohort had average activity scores that indicated a minimum functional level of moderately heavy labor and recreational sports, such as cycling, cross-country skiing, and/or jogging, on even ground at least twice weekly [13].

### Survivorship

All-cause survivorship reported at 10 years or less ranged from 90.6% to 99.0%, with LR studies averaging 96.9% and the LS cohort averaging 97%. From 11 to 20 years, survivorship ranged from 83.9% to 96.5%, with LR studies averaging 87.3% and the LS cohort averaging 94%. The LS cohort studies are the only of their kind to report on TKA survivorship greater than 20 years for the same cohort. In these studies, survivorship reported at 25.1 years ranged from 70.1% to 70.6%. At the current stage of the study of the LS cohort, survivorship at 40 years has an upper limit of 65.3% and a lower limit of 52.1%, accounting for the difficulty in contacting the LS patient population for follow-up.

In the LS cohort, when examining for tibial or femoral component aseptic failure, there was a significant difference in survivorship between the non-modular Insall-Burstein I component and the modular Insall-Burstein II component at 25-year follow-up, at 92.3% and 68.3%, respectively [13].

### Radiolucency

Five studies reported data on radiolucency. No progressive radiolucency was reported in any of the studies. Diduch et al. and Keeney et al. found non-progressive radiolucent lines in 9% and 14.5% of cases, respectively. Mont et al. found radiolucent lines less than 1 mm in 13.3% of cases, and 1–2 mm in 10% of cases. No progressive radiolucencies were reported in both Long et al. and Odland et al.

### Risk factors for revision

Aujla et al. Old et al. Karas et al. and Camus et al. all observed that female patients under age 55 experienced a higher rate of revision [7, 10, 16, 17].

The authors of studies in which both cemented and cementless designs were used generally found no statistically significant difference in the risk for future revision [10, 16].

Camus et al. found a significant increase in revision rate when the Insall-Burstein II implant (Zimmer, Warsaw IN) was used, compared to use of the Insall-Burstein I (Zimmer, Warsaw IN) or Constrained Condylar (Zimmer, Warsaw IN) implants [8]. The Insall-Burstein II implant was associated with an even greater risk of revision when thinner polyethylene was used. Further, they found that revision rate was higher with patients with greater activity levels after their index TK [7].

### Revision etiologies

Nine of the 10 studies included information on the most prevalent risk factors for revision. In 6 of the studies, aseptic loosening was cited as the most common reason for revision [6, 7, 10, 12, 16, 17]. Other prevalent reasons for revision included infection, polyethylene wear, patellar component failure, and instability [10, 12, 17–20].

### Outcomes of revised TKAs In the LS cohort

In the LS cohort, outcomes of revision TKAs were assessed at a mean of 10.5 years after revision, at a mean of 25.1 years after the index primary TKA. The average KS clinical and functional scores were 89.0 and 75.6, respectively. The revised cohort’s mean Tegner activity score of 4.6 exceeded that of unrevised TKAs from the same original cohort, of which the mean was 2.9. Average ROM was 119°, and analysis of radiographs showed no evidence of component loosening in any revised knees [17].

## Discussion

As the incidences of osteoarthritis and resulting TKAs continue to rise among younger adults, there is a need for data on long-term outcomes and survivorship. The aim of this study was to review the recent literature on total joint replacement for the management of end-stage osteoarthritis in patients aged 55 or younger and summarize their collective reported outcomes.

TKA in younger patients has been a controversial topic because surgeons must strike a delicate balance between providing relief for patients with end-stage osteoarthritis and placing an implant that may degenerate from wear, requiring revision over the remainder of the patient’s life [21]. For this reason, younger TKA patients frequently have higher expectations for their return to activity and have often reported lower satisfaction rates [21, 22]. However, in the present study’s young patient population, range of motion and functional scores improved postoperatively at all postoperative time points studied, owing to the long-lasting positive functional impact of TKA for osteoarthritic patients. The results obtained from the LS cohort post-TKA were very similar to those of the LR cohort, none of which had average follow-up periods past 20 years. Therefore, these results support the conclusion that the LS cohort’s findings at 30 and 40 years post-TKA are adequately representative of the average young, active TKA patient. Further, the favorable and durable clinical outcomes seen in the LS cohort’s revised patients show that young, osteoarthritic patients may benefit from TKA, even if they later require revision.

A unique advantage of studying TKA outcomes in young patients is the ability to study implant survivorship in a way that cannot be appreciated in the typically older patient population. TKA survivorship in patients older than 55 has been consistently strong, with several studies reporting 95% survivorship or greater at 10 years [23, 24]. Our review found that survivorship in young patients ranged from 90.6% to 99.0% at 10 years, which is generally a wider range than what is reported for older patients. This phenomenon could be explained by a variety of factors, the most likely being that younger patients are more active and are therefore more likely to wear out the implant [25, 26]. This is reflected in our finding that the most common cause for revision across all studies was aseptic loosening, a widely known product of excessive wear [25, 26]. However, aseptic loosening as a cause for revision has decreased dramatically in recent years, in large part due to improvements in implant design and diminished utilization of implants prone to wear, such as the Insall-Burstein II design [27].

These trends continue into the studies with longer follow-up periods, and it is important to collect data on longer follow-up periods to assess TKA implant effectiveness and longevity. Previous research has reported on lifetime TKA revision risk, as an assessment of the New Zealand Joint Registry database reported a 22.4% lifetime revision risk in patients aged 46–50, but the average follow-up was only 18 years [28]. Therefore, our research group recently began gathering survivorship data on the LS cohort at 40 years. Accounting for difficulties with data collection on a cohort of patients where the majority were deceased or lost to follow-up, and following a global pandemic, we determined that 40-year survivorship could fall in the range of 52.1–65.3%. It is significant that over half of our patients retained their original implant, which is a testament to the durability of TKA over a 40-year time period. This finding also has implications for informed shared decision-making between arthroplasty surgeons and younger patients considering TKA. Although the majority of original implants lasted in the LS cohort, young patients with high activity levels, and who plan to sustain such high activity through 40 years post-TKA should still consider the possibility of needing a revision later in life. The 40-year follow-up data collected by our research group is still an ongoing area of investigation, and more detailed outcomes will be reported in future literature.

A significant finding outside of the TKA outcomes themselves was the scarcity of which this topic is studied. Even with the relatively broad inclusion criteria of studies including osteoarthritic patients under 55 with primary TKA, our literature search only yielded six studies outside of the follow-up studies on our own LS cohort. Because TKA is becoming increasingly popular and its indications are expanding to include more young patients, it is imperative that these patient populations are adequately studied in order for them to be properly informed about long-term outcomes prior to committing to surgery [29]. The American Joint Replacement Registry is a vital resource for this purpose, and as it expands, it will provide an increasing wealth of data on modern implants and techniques in young patients undergoing TKA [27].

Our study has many strengths, most notably that it contains the longest-term TKA survivorship data in young patients ever studied. Further, it includes follow-up data on revised TKAs and their outcomes, providing a more comprehensive understanding of the global expectation to a young patient considering TKA. However, this study should also be interpreted in the context of its limitations. There were few studies that met the inclusion criteria, decreasing the sample size from which we could draw conclusions about TKA outcomes in young patients. Not every study reported all of our outcome measures, further decreasing that sample size. Studies that only reported survivorship for specific modes of implant failure were excluded from our survivorship analysis, which encompassed all-cause revision. Within just the LS cohort, there was a small sample size, and many patients were lost to follow-up due to difficulties with contacting patients using their listed contact information, decreasing the accuracy with which we could report survivorship. This is the reason that we elected to report a survivorship range instead of an exact percentage for the 40-year follow-up cohort.

## Conclusion

In this review of the literature on TKA outcomes in osteoarthritic patients under age 55, we found that patients had durable, improved functionality at each follow-up time point assessed. There was greater variation in 10-year survivorship among these younger patient studies compared to published survivorship figures from older patient populations. Survivorship decreased with increasing lengths of follow-up, reaching a minimum in the range of 52.1–65.3% at 40 years post-TKA. The scarcity of literature on long-term TKA outcomes in this patient population speaks to the necessity of further research on this topic.
